# Supplementary material for: Uveal melanoma: Long-term survival
Source: PLoS One. 2021 May 18;16(5):e0250939. doi: 10.1371/journal.pone.0250939 (PMC8130945; doi:10.1371/journal.pone.0250939)
Supplement: S1 Text — (PDF) [file pone.0250939.s001.pdf]

S1. TEXT. R Scripts Used in "Uveal Melanoma: Long-Term Survival"

Tomas Radivoyevitch, Emily C. Zabor, and Arun D. Singh

Quantitative Health Sciences, Lerner Research Institute, and
Ophthalmic Oncology, Cole Eye Institute, Cleveland Clinic, Cleveland, USA

SEER data can be requested **at** https://seer.cancer.gov/data/access.html

SEER no longer offers ASCII data needed, but if you have 1975-2016 ASCII treatment data, follow https://github.com/radivot/SEERaBomb, and include the field primsite using

```
picks=c("casenum","reg","race","sex","agedx",
        "yrbrth","seqnum","modx","yrdx","histo3",
         "ICD9","primsite","COD","surv","radiatn","chemo")
(rdf=pickFields(df,picks))
mkSEER(rdf,outFile="cancPrim",writePops=F)
```

you should be able to run

```
load("~/data/SEER/mrgd/cancPrim.RData")#load SEER cancer data
d=canc%>%filter(primsite%in%c("C693","C694","C692"),histo3%in%8720:8790)
d$cancer="uveal"
d=d%>%filter(!is.na(surv))%>%mutate(status=as.numeric(COD>0),surv=(surv+0.5)/12)
d5=d%>%select(yrdx,agedx,sex,surv,status)
setwd("~/case/active/seer/uveal/seerEAR")
save(d,d5,file="data/d.RData")
```

and make the Fig 2 plots as follows:

First the OS/MFS plot (Fig. 2A) and RS plots (Fig. 2C-D)

```
graphics.off();rm(list=ls())#clear plots and environment
library(SEERaBomb);library(tidyverse)
library(survival);library(survminer)
load("data/d.RData")
load("data/mrtUSA.RData") #loads US mortality data
gp=geom_point(); gl=geom_line()
ge=geom_errorbar(aes(ymin=LL,ymax=UL),width=0.2)#for absolute risks
sbb=theme(strip.background=element_blank())
ltb=theme(legend.margin=margin(0,0,0,0),legend.title=element_blank())
ltp=theme(legend.position="top")
lh=theme(legend.direction="horizontal")
tc=function(sz) theme_classic(base_size=sz);
svts=scale_x_continuous(breaks=seq(0,35,5),limits=c(0,38))
gx=xlab("Years Since Diagnosis")
gy=ylab("Excess Absolute Risk of Mortality")
```

```
library("MALDIquant")
library(WriteXLS)
(d6=d%>%select(yrdx,agedx,sex,surv,status,CODS))
d6=d6%>%mutate(status=ifelse((status==1)&(!CODS%in%c("melanoma","eye")),0,status))
(d6=d6%>%select(-CODS))
d5$grp="All Cause Mortality"
d6$grp="Death by Melanoma"
D=bind_rows(d5,d6)
fit=survfit(Surv(surv,status)~grp,data=D)
gy=ylab("Survival Probability")
labs=c("OS","MFS")
agts=scale_x_continuous(breaks=seq(0,40,5))#age times
ggsurvplot(fit,D,legend.title="",legend.labs=labs)+gx
ggsave("outs/survOSnMFS.pdf",width=4.5,height=3) #Fig. 2A
ggsurvplot(fit,D,legend.title="",legend.labs=labs,
           cumevents=T)+gx
cumevents=T,risk.table=T)+gx
s=summary(fit)
round(c(tail(s$surv)[6],tail(s$lower,1),tail(s$upper,1)),3)
# # #so MFS yields 60% (0.584 0.629) # no sex diffs commented
# fitS=survfit(Surv(surv,status)~grp+sex,data=D)
# labs=c("OSmale","OSfem","MFSmale","MFSfemale")
# ggsurvplot(fitS,D,legend.title="",legend.labs=labs)+gx
# ggsave("uveal/outs/survOSnMFSbySex.pdf",width=4.5,height=3)
tb=tibble(t=s$time,P=s$surv,LL=s$lower,UL=s$upper,grp=s$strata)
table(tb$grp)
tbOS=tb%>%filter(grp=="grp=All Cause Mortality")
tbMFS=tb%>%filter(grp=="grp=Death by Melanoma")
tos=tbOS$t
tmfs=tbMFS$t
x=seq(5,35,5)
(kos=match.closest(x,tos))
mkCI=function(P,LL,UL)
  paste0(sprintf('%.3f',P)," (",sprintf('%.3f',LL),", ",sprintf('%.3f',UL),")")
(tbOSk=tbOS[kos,]%>%mutate(CI=mkCI(P,LL,UL)))
(kmfs=match.closest(x,tmfs))
(tbMFSk=tbMFS[kmfs,]%>%mutate(CI=mkCI(P,LL,UL)))
L=list(KMtable=tibble(Time=x,
                      "KM OS Probability"=tbOSk$CI,
                      "KM MFS Probability"=tbMFSk$CI
))
WriteXLS(L,ExcelFileName="outs/KMestimates.xlsx",AdjWidth = T) #table 2
tbMFSk%>%mutate(mCI=mkCI(1-P,1-LL,1-UL)) #1-MFS values for text

ddT=simSurv(d,mrt,rep=3,ltb=NULL,unif=TRUE)  # takes several seconds
fit=survfit(Surv(surv,status)~type,data=ddT)
labs=c("Observed","Expected")
ggsurvplot(fit,ddT,legend.title="",legend.labs=labs,xlim=c(0,60))+gx
ggsave("outs/survWithCntrl.pdf",width=4.5,height=3)


fitd=survfit(Surv(surv,status)~1,data=ddT%>%filter(type=="Observed"))#d=data
fitc=survfit(Surv(surv,status)~1,data=ddT%>%filter(type=="Simulated"))#c=control
x=seq(0,42,.1)
(tc=fitc$time)
```

```
(kc=match.closest(x,tc))
Dc=tibble(time=fitc$time,surv=fitc$surv,LL=fitc$lower,UL=fitc$upper)
Dc=Dc[kc,]
# (svc=fitc$surv[kc])

(td=fitd$time)
(kd=match.closest(x,td))
# Dd=tibble(time=fitd$time,surv=fitd$surv,LL=fitd$lower,UL=fitd$upper)%>%slice(kd)
Dd=tibble(time=fitd$time,surv=fitd$surv,LL=fitd$lower,UL=fitd$upper)
Dd=Dd[kd,]
# Dd
# tail(Dd)
# (svd=fitd$surv[kd])
gy=ylab("Relative Survival")
gh=geom_hline(yintercept=0.6)
agy=scale_y_continuous(breaks=seq(0,1,.2),limits=c(0,1))#age times
gtc=function(sz) theme_classic(base_size=sz);
pRS=tibble(x,svr=Dd$surv/Dc$surv)
pRS%>%ggplot(aes(x,svr))+geom_step()+gx+gy+gh+agy+gtc(14)
# tibble(x,svr=svd/svc)%>%ggplot(aes(x,svr))+geom_step()+gx+gy+gh+agy+gtc(14)
ggsave("outs/survRel.pdf",width=4.5,height=3)
save(pRS,file="data/pRS.RData")

pRSci=tibble(x,svr=Dd$surv/Dc$surv,LL=Dd$LL/Dc$surv,UL=Dd$UL/Dc$surv)
pRSci%>%ggplot(aes(x,svr))+geom_step()+
  geom_line(aes(x,UL),col="gray")+
  geom_line(aes(x,LL),col="gray")+gx+gy+gh+agy+gtc(14)
ggsave("outs/survRelCI.pdf",width=4.5,height=3)


x0=seq(5,35,5)
(kc5=match.closest(x0,Dc$time))
Dc5=Dc[kc5,]
Dc5

(kd5=match.closest(x0,Dd$time))
# Dd5=Dd%>%slice(kd5)
Dd5=Dd[kd5,]
Dd5$time=x0
(dRS=Dd5%>%mutate_at(vars(surv:UL),function(x) x/Dc5$surv))
dRS=dRS%>%mutate(CI=mkCI(surv,LL,UL))
L=list(RStable4=dRS%>%select(Time=time,
                             "Relalite Survival"=CI))
WriteXLS(L,ExcelFileName="outs/RelSurvTable.xlsx",AdjWidth=T)
```

Now the competing risks plot (Fig. 2B)

```
library(cmprsk) #run after surv.r
(d=d%>%select(yrdx,agedx,sex,surv,status,CODS))
D2=d%>%mutate(status=ifelse((status==1)&(!CODS%in%c("melanoma","eye")),2,status))
fitc=with(D2,cuminc(surv,status))
labs=c("Death by Melanoma","Death by Other Causes")
```

```
names(fitc)=c(" 1"," 2")  # gets rid of 1 at top
(g=ggcompetingrisks(fitc)+sbb+gx+ylab("Cumulative Probability of Death")+
    scale_color_manual(labels=labs,values=c("#F8766D","#00BFC4"))+ylim(c(0,1))+
    theme(legend.position = c(0.3, 0.8),legend.title=element_blank())
+labs(title="",caption=""))
ggsave("outs/cumIncY0to1.pdf",width=4.5,height=3.5) #Fig. 3
library("MALDIquant")
dmfs=as_tibble(fitc[[1]])
dos=as_tibble(fitc[[2]])
x0=seq(5,35,5)
(kos=match.closest(x0,dos$time))
(dos=dos[kos,]%>%mutate(CIos=paste0(sprintf('%.3f',est)," (",
                                    sprintf('%.3f',est-1.96*sqrt(var)),", ",
                                    sprintf('%.3f',est+1.96*sqrt(var)),")")))
(kmfs=match.closest(x0,dmfs$time))
(dmfs=dmfs[kmfs,]%>%mutate(CImfs=paste0(sprintf('%.3f',est)," (",
                                 sprintf('%.3f',est-1.96*sqrt(var)),", ",
                                 sprintf('%.3f',est+1.96*sqrt(var)),")")))

L=list(CumInc=tibble(Time=x0, "All Death Cumulative Incidence"=dos$CIos, #table 3
                     "Melanoma Death Cumulative Incidence"=dmfs$CImfs))
WriteXLS(L,ExcelFileName="outs/cumIncEstimates.xlsx",AdjWidth = T)
```

and now the EAR (Fig. 2E) and overall comparison (Fig 2F) plots

```
quantile(d$surv)
(d=d%>%select(yrdx,agedx,sex,surv,status,CODS))
(D1=d%>%mutate(status=ifelse((status==1)&(!CODS%in%c("melanoma","eye")),0,status)))
fit=survfit(Surv(surv,status)~1,data=D1)

dkm=tibble(t=fit$time,y=1-fit$surv,Method="Kaplan-Meier")
(D2=d%>%mutate(status=ifelse((status==1)&(!CODS%in%c("melanoma","eye")),2,status)))
fitc=with(D2,cuminc(surv,status))
dci=tibble(t=fitc[[1]]$time,y=fitc[[1]]$est,Method="Competing Risks")
(Ds=msd(d5,mrt,brkst<-c(0,0.5,1,2,3,4,5,6,8,10,12,15,20,25,30,35)))
Ds=Ds%>%rename(Group="sex")%>%select(Group,int,everything()) #%>%arrange(Group,t)
D1=foldD(Ds)%>%mutate(Group="Pooled")%>%select(int,Group,everything())

(levs=levels(D1$int))
paste0(levs,collapse=", ")
(tb=as_tibble(t(sapply(levs,function(x) getBinInfo(x[1],x)))))
(tb=tb%>%mutate(delta=UL-LL))
range(d$surv)
tb$delta[16]=7
tb$ear=D1$EAR
tb$t=D1$t
(tb=tb%>%mutate(prod=delta*ear,cprod=cumsum(prod),
                surv=exp(-cprod),y=1-surv,
                Method="1-exp(-sum(EAR))"))
t=seq(0,40,.01)
mylin=function(t,m1=0.0011,t1=14.4,t2=26.8) {
  y=ifelse(t<t1,m1*t,0)
  y=ifelse((t>=t1)&(t<t2),m1*t1-(m1*t1/(t2-t1))*(t-t1),y)
  y=ifelse(t>=t2,0,y)
```

```
  y
}
mygam=function(t,c=-0.37849645,k=0.39624240) k^2*(t/2)*exp(c-k*t)
pd=tibble(t,Lines=mylin(t),Gamma=mygam(t),Sum=Lines+Gamma)
pd=pd%>%gather(key="func",value="y",-t)

pS=pd%>%filter(func=="Sum")
pS=pS%>%mutate(cy=cumsum(0.01*y),y=1-exp(-cy),Method="Excess Absolute Risk")

library(bbmle)#O=EAR*PY+E
(s=summary(mod<-mle2(O~dpois(lambda=(mygam(t,c,k)+mylin(t,m1,t1,t2))*PY+E),
                     method="Nelder-Mead",
                     start=list(c=0,k=1,m1=0.002,t1=10,t2=20),data=D1,
                     control=list(maxit=10000))))
library(broom)
mkCIm=function(est,sd) paste0(sprintf('%.4f',est)," (",
                              sprintf('%.4f',est-1.96*sd),", ",
                              sprintf('%.4f',est+1.96*sd),")")
(params=tidy(mod)%>%mutate(CI=mkCIm(estimate,std.error)))

library(WriteXLS)
WriteXLS(list(Table5=params%>%select(Parameter=term,    #table 5
                                     "Estimate (95% CI)"=CI,
                                     "P Value"=p.value)),
         ExcelFileName="outs/TableModParams.xlsx",AdjWidth=T)
# save(mod,file="data/earFit.RData")

pd=pd%>%mutate(comp=ifelse(func=="Lines","Triangle",func),func=as_factor(comp))
D1%>%ggplot(aes(x=t,y=EAR))+gp+gx +
  geom_line(aes(x=t,y=y,col=func),data=pd)+
  tc(14)+ltb+ltp
ggsave("outs/poisRegEARfig2E.pdf",width=4,height=3)

load("data/pRS.RData")
drs=pRS%>%mutate(t=x,y=1-svr,Method="Relative Survival")
# D=bind_rows(list(dkm,dci,drs,tb%>%select(t,y,Method),pS%>%select(t,y,Method)))
D=bind_rows(list(dkm,dci,drs,pS%>%select(t,y,Method)))
D%>%ggplot(aes(t,y,col=Method))+geom_step()+gx+
  ylab(" Probability of Uveal Melanoma Death")+tc(13)+ltb+
  theme(legend.position=c(.6,.28)) +
  # scale_colour_manual(name = "Method",
  #  values = c("red","orange","green","blue", "violet"),
  #  labels = expression(exp(-integral(hds)),exp(-sum(EAR)),1-MFS,1-
RS,CompetingRisk))
ggsave("outs/comparisonFig2F.pdf",width=4.5,height=3.5)
```

The remaining code is for miscellaneous calculations

Fig. 1

```
load("data/allfields2019.RData")
library(dplyr)
d$CODS=as.character(d$CODS)
```

```
d=d%>%mutate(CODS=ifelse(!CODS%in%c("alive","melanoma","eye"),"other",CODS))
d$CODS[d$CODS=="eye"]="melanoma"
d=d%>%mutate(status=as.numeric(COD>0),surv=(surv+0.5)/12)
d=d%>%mutate(survG=cut(surv,c(0,5,10,15,20,25,30,45),
                       dig.lab=4,include.lowest=T,right=F))
m=d
table(m$survG,m$CODS) # make this in dplyr
library(tidyr)
(fg1=m%>%group_by(survG,CODS)%>%summarize(n=n()))
(fg1=fg1%>%spread(CODS,n))
# (fg1=fg1%>%mutate(n=other+melanoma))
# (fg1=fg1%>%mutate(frc=other/n))
# fg1%>%group_by()%>%summarize(M=sum(melanoma),O=sum(other),P=O/(M+O))
library(WriteXLS)
WriteXLS(list(Fig1=fg1),ExcelFileName="outs/fig1.xlsx")


#fraction of deaths by UVM in all vs SEER9
(tb=table(d$CODS))
sum(tb[2:3])
tb[2]/sum(tb[2:3])
d9=d%>%filter(db=="73")
(tb=table(d9$CODS))
sum(tb[2:3])
tb[2]/sum(tb[2:3])
table(d$db)

d9=d9%>%filter(yrdx<1987)
(tb=table(d9$CODS))
sum(tb[2:3])
tb[2]/sum(tb[2:3])
tb[2]/sum(tb)
```

Table 1 using's SEERaBombs mkDemographics()

```
load("~/data/SEER/mrgd/cancPrim.RData")#load SEER cancer data
load("~/data/SEER/mrgd/popsae.RData")#load SEER population data
head(canc,2)
canc=canc%>%filter(primsite%in%c("C693","C694","C692"),histo3%in%8720:8790)
canc$cancer=as.character(canc$cancer)
# C69.3 (choroid), C69.4 (ciliary body and iris), and C69.2 (retina).
canc$cancer[canc$primsite=="C693"]="Choroid"
canc$cancer[canc$primsite=="C694"]="Ciliary"
canc$cancer[canc$primsite=="C692"]="Retinal"
canc%>%filter(is.na(surv))%>%print(n=4)
# one choroid survival is missing so tables will be shy 1 for it
d=canc%>%filter(!is.na(surv))%>%print(n=4)
d$age=cut(d$agedx,c(0,40,60,80,126),include.lowest = T)
d$year=cut(d$yrdx,c(1975,1995,2016),include.lowest = T,dig.lab=4)
mkDemographics(d,outDir="outs")
d$cancer="uveal"
mkDemographics(d,outDir="outs")
```

If you do not have 1975-2016 ASCII treatment data, summary data needed to produce Fig. 2E is provided on GitHub in D1.RData here. Fig2E can be reproduced by applying to this data the script Fig2Ealone.R found on Github here.
